# Indication‐specific event rates among hospitalized patients undergoing continuous cardiac monitoring

**DOI:** 10.1002/clc.23244

**Published:** 2019-08-12

**Authors:** Daniel J. Cantillon, Alicia Burkle, Desiree Kirkwood, Molly Loy, Ram Amuthan, Shannon Pengel, John Tote, William Morris, Penny L. Houghtaling, Aaron C. Hamilton, Marc Petre, Umesh N. Khot, Bruce D. Lindsay

**Affiliations:** ^1^ Heart and Vascular Institute Cleveland Clinic Cleveland Ohio; ^2^ Nursing Institute Cleveland Clinic Cleveland Ohio; ^3^ Internal Medicine/Hospital Medicine Cleveland Clinic Cleveland Ohio; ^4^ Lerner Research Institute Cleveland Clinic Lerner College of Medicine Cleveland Ohio; ^5^ Community Medicine/Hospital Medicine Cleveland Clinic Cleveland Ohio; ^6^ Clinical Engineering Cleveland Clinic Cleveland Ohio

**Keywords:** alarm fatigue, arrhythmias, cardiac telemetry, monitoring

## Abstract

**Background:**

Cardiac telemetry monitoring is widely utilized for a variety of clinical indications, yet indication‐specific event rates for monitored patients are seldomly reported.

**Hypothesis:**

High‐risk hospitalized patients for clinical deterioration can be identified using standardized telemetry monitoring indications.

**Methods:**

Adjudicated data from events triggering emergency response team (ERT) activation were systematically characterized at the Cleveland Clinic from among standardized telemetry indications ordered over a 13‐month period.

**Results:**

Among 72 199 orders created for telemetry monitored patients, ERT activation occurred in 2677 patients (3.7%), of which 1326 (49.5%) were cardiac‐related. Patients with deep venous thrombosis or pulmonary embolism (DVT/PE) demonstrated the highest overall event rate (ERT: n = 41 of 593 pts [6.9%]; 25/41 cardiac related [61%]). Cardiac‐related events were proportionally highest among patients with coronary disease awaiting revascularization (ERT: n = 19 of 847 patients [2.2%]; 13/19 cardiac‐related [68.4%]). Arrhythmia‐specific events were highest among patients who underwent cardiac surgery (n = 78 of 193 cardiac‐related ERT [40.4%]), and patients with known or suspected tachyarrhythmias (n = 318 of 788 cardiac‐related ERT [40.4%]). Bubble plot analysis identified patients hospitalized with DVT/PE, drug or alcohol exposures, and acute coronary syndrome as among the highest overall and cardiac‐related events while identifying patients with respiratory disorder monitoring indications as carrying the highest noncardiac event rate.

**Conclusion:**

High‐risk hospitalized patients can be identified by telemetry indication and prioritized according to concerns for cardiac, arrhythmia‐specific and noncardiac clinical deterioration. This is particularly useful when monitored bed resources are constrained.

## 1 INTRODUCTION

Cardiac telemetry monitoring is widely utilized for a variety of clinical indications, yet indication‐specific event rates for monitored patients are seldomly reported.^1^, ^2^, ^3^, ^4^, ^5^ The 2017 update to American Heart Association guidelines^6^ call for additional research while endorsing standardized hospital‐based monitoring practices.^7^ Standardized cardiac telemetry monitoring, in accordance with practice guidelines, has been associated with decreased utilization, cost savings, and improved clinical outcomes.^1^, ^2^ Removing low‐risk patients may mitigate alarm fatigue by reducing the volume of inactionable alarms.^3^, ^4^, ^5^, ^8^ In the ALARMED study, cardiac telemetry findings altered management in only 0.2% of low‐risk patients admitted for chest pain despite generating an average 4.7 alarms per hour.^5^ Delineating the indication‐specific event rates for cardiac, noncardiac, and arrhythmia‐specific clinical deterioration is therefore needed to improve risk stratification and prioritization, particularly when monitored bed resources are constrained. This aligns with patient safety goals set forth by the Joint Comission.^9^ The present analysis therefore examined indication‐specific adjudicated event rates during a previously reported 13‐month period of applying standardized telemetry monitoring indications with an electronic order linked to offsite central monitoring.^1^

## 2 METHODS

Previously published standardized telemetry monitoring indications were systemically captured for noncritically ill hospitalized patients at the Cleveland Clinic using required electronic order entry over a 13‐month period (4 March 2014 to 4 April 2015), as listed in Table 1.^1^ Emergency response team (ERT) activations were systematically and simultaneously collected as an endpoint for clinical deterioration, and were adjudicated into cardiac and noncardiac groups with additional substratification of arrhythmia‐specific events. Noncardiac ERT activations included altered mental status, as well as symptoms or clinical changes attributed to a noncardiovascular cause (ie, bleeding, sepsis, noncardiac respiratory failure). Nonarrhythmic cardiac ERT activations included symptoms, vitals‐based or ECG changes attributed to heart disease including ischemia. Arrhythmia‐specific cardiac events included ERT triggered by bradyarrhythmias or tachyarrhythmias. The ERT consists of a dedicated physician, nurse, and respiratory therapist with rapid deployment capability and authorization to provide temporary management or facilitate intensive care unit (ICU) transfer and is available 24 hours/day, 7 days/week. Per policy, ERT activation is triggered by bedside personnel including nursing, physicians, or advanced practice providers for clinical concerns requiring urgent and immediate evaluation and/or treatment. The systematic collection and categorization of ERT events was adjudicated by review of clinical documentation, including all rhythm strips 1 hour prior to the event by an independent outcomes coordinator, and physician medical director. Categorical variables were expressed as numbers and percentages. Data collection, analysis, and reporting were performed in REDCap (Version 5.8.2, 2015, Vanderbilt University). Patient risk stratification for both cardiac and noncardiac ERT responses was performed using a bubble plot methodology capturing the number of orders for a given indication (volume), the percentages of overall ERT activations and those that are cardiac‐related. The study database carries Institutional Review Board approval for retrospective analysis intended for research and quality assurance purposes with an informed consent waiver for de‐identified patient data analysis and reporting.

**Table 1:** Standardized cardiac telemetry indications utilized at Cleveland Clinic during the study period

1	Atrial or ventricular tachyarrhythmias, known or suspected
2	Bradycardia, known or suspected including sinus node dysfunction, Atrioventricular (AV) block
3	Bradycardia: Temporary pacing with escape rate greater than 40 bpm
4	Cardiac surgery, postoperative
5	Cardiac electrophysiology procedure (pacer, Implantable cardioverter defibrillator (ICD), ablation)
6	Cardiac or endovascular intervention, percutaneous (Percutaneous coronary intervention (PCI), structural, vascular)
7	Coronary disease: Acute coronary syndrome, known or suspected (“rule out MI”)
8	Coronary disease: High‐risk awaiting revascularization
9	Deep venous thrombosis/pulmonary embolism (DVT/PE)
10	Drug/alcohol exposure (cocaine, amphetamines, opiates, and so forth)
11	Heart failure: Acutely decompensated
12	Heart failure: Chronic/subacute
13	Hospital transfer within 72 h
14	Long QT syndrome or channelopathy
15	Metabolic derangement (acid/base, electrolyte, glycemic disturbance)
16	Moderate sedation procedure (ie, Transesophageal echocardiogram (TEE), endoscopy, diagnostic angiograms, Interventional radiology (IR))
17	Other: Free text response
18	Palliative: Terminal illness with arrhythmias causing discomfort
19	Pro‐arrhythmic drug therapy
20	Respiratory disorder, acute (Chronic obstructive pulmonary disease (COPD), Obstructive sleep apnea (OSA), pneumonia)
21	Seizure monitoring and/or anti‐epileptic therapy
22	Stroke or transient ischemic attack (TIA)
23	Syncope/presyncope, evaluation or treatment

## 3 RESULTS

There were 72 199 telemetry orders placed at the Cleveland Clinic representing a de novo order for a continuously monitored patient for up to 72 hours hospitalization, with a renewal order required after that time. There were 2677 ERT activation events (3.7%), of which 1326 (49.5%) were cardiac‐related. The complete list of indication‐specific utilization and with overall ERT event rates is shown in Table 2, and by cardiac‐related events in Table 3. The most common telemetry indication by volume was for patients whom underwent cardiac surgery (n = 14 426 [20.0%]), of which there were 193 ERT activations (1.3%) including 93 (48.2%) cardiac‐related, and 78 (40.4%) rhythm‐related events. The two least commonly utilized indications included palliative care in which arrhythmias were known to cause patient discomfort (n = 29 [<1%]; ERT: n = 3 [10.3%]) and bradycardia with temporary pacing (n = 98 [<1%]; ERT: n = 1 [1.0%]) where no cardiac‐related events occurred. The highest cardiac‐related event rate occurred among patients with coronary disease awaiting revascularization (n = 848 [1.2%]; ERT: n = 19 [2.2%]; 13/19 cardiac‐related [68.4%]). The highest arrhythmia‐specific event rate occurred among patients who underwent cardiac surgery (n = 78/193 rhythm‐related [40.4%]), and patients with the known or suspected atrial or ventricular tachyarrhythmias (n = 318/788 rhythm‐related [40.4%]). Both of these indications were dominated by supraventricular arrhythmias (cardiac surgery: n = 58/78 [74.4%]; known/suspected tachyarrhythmias: n = 292/415 [70.4%]). For noncardiac events, the respiratory disorder category (n = 1511 [2.1%]) had the highest rate (ERT: n = 85 [5.6%]; 57/85 noncardiac related [67.1%]).

**Table 2:** Telemetry indication‐specific emergency response team (ERT) activation event rates listed in descending order (first column) along with the corresponding orders volume (second column)

Indication	ERT events (%)	Volume (%)
Deep venous thrombosis/pulmonary embolism	41 (6.9%)	593 (1%)
Atrial or ventricular arrhythmias	788 (6.7%)	11 785 (16%)
Drug exposure	20 (6.1%)	328 (<1%)
Syncope	64 (5.7%)	1123 (2%)
Respiratory disorders	85 (5.6%)	1511 (2%)
Long QT syndrome	13 (5.4%)	239 (<1%)
Metabolic derangement	193 (4.6%)	4156 (6%)
Bradycardia/AV block	57 (4.6%)	1249 (2%)
Acute coronary syndrome	181 (4.5%)	4037 (6%)
Other category: free texted^a^	411 (4.2%)	9799 (14%)
Seizure monitoring	80 (3.1%)	2567 (4%)
Postprocedure: moderate sedation	57 (3.5%)	1652 (2%)
Heart failure: chronic	128 (3.3%)	3862 (5%)
Stroke/transient ischemic attack	63 (3.2%)	1949 (3%)
Hospital transfer	104 (3.0%)	3512 (5%)
Pro‐arrhythmic drug therapy	34 (3.2%)	1052 (1%)
Coronary artery disease: awaiting revascularization	19 (2.2%)	848 (1%)
Heart failure: acutely decompensated	94 (2.1%)	4462 (6%)
Postprocedure: electrophysiology	24 (2.5%)	976 (1%)
Bradycardia: temporary pacing wire	1(1.0%)	98 (<1%)
Cardiac surgery	193 (1.3%)	14 426 (20%)
Postprocedure: interventional cardiology	24 (1.2%)	1946 (3%)
Palliative care	3 (10.3%)	29 (<1%)

a Other category: The largest group within this category was “hypotensive disease states” that included GI bleeding, sepsis/bacteremia, and pancreatitis (n = 607; <1%) with 40 ERT activations (6.6%) including 10 cardiac (25%), and eight rhythm‐related (20%).

**Table 3:** Cardiac‐related emergency response team (ERT) event rates listed by telemetry indication in descending order occurring as a percentage of the total number of events (first column) and with the corresponding overall ERT event rate (second column) occurring as a percentage of the order volume

Indication	Cardiac‐related ERT events (%)	Total ERT events (%)
Coronary artery disease: awaiting revascularization	13 (68.4%)	19 (2.2%)
Rhythm‐specific	3 (15.8%)	
Stroke/TIA	40 (63.5%)	63 (3.2%)
Rhythm‐specific	22 (34.9%)	
DVT/PE	25 (61%)	
Rhythm‐specific	11 (26.8%)	41 (6.9%)
Acute coronary syndrome	108 (59.7%)	181 (4.5%)
Rhythm‐specific	42 (23.2%)	
Drug exposure	12 (60%)	20 (6.1%)
Rhythm‐specific	6 (30%)	
Bradycardia/AV block	31 (54.4%)	57 (4.6%)
Rhythm‐specific	17 (29.8%)	
Moderate sedation	31 (54.4%)	57 (3.5%)
Rhythm‐specific	19 (33.3%)	
Long QT syndrome	7 (53.8%)	13 (5.4%)
Rhythm‐specific	3 (23.1%)	
Pro‐arrhythmic drug therapy	18 (52.9%)	34 (3.2%)
Rhythm‐specific	9 (26.5%)	
Atrial/ventricular tachyarrhythmias	415 (52.7%)	788 (6.7%)
Rhythm‐specific	318 (40.4%)	
Seizure monitoring	42 (52.5%)	80 (3.1%)
Rhythm‐specific	17 (21.3%)	
Hospital transfer	53 (51%)	104 (3.0%)
Rhythm‐specific	36 (34.6%)	

*Note*: The rhythm‐specific event rates occurring as a percentage of cardiac‐related events in a separate row beneath each indication.

Noncardiac indications for telemetry monitoring included patients with metabolic derangement (n = 4156 [5.8%]), respiratory disorders (n = 1511 [2.1%]), seizure monitoring (n = 2567 [3.6%]), stroke/transient ischemic attack evaluation (n = 1949 [2.7%]), deep vein thrombosis/pulmonary embolism (n = 593 [<1%]), and drug/alcohol exposures (n = 328 [<1%]). Among these, the highest overall event rate occurred among patients with metabolic derangements including 193 ERT activations (4.6%), of which 89 were cardiac‐related (46.1%), with 56 of these being rhythm‐related events (29.0%). Among drug/alcohol exposures, 13 of the 22 ERT activations were cardiac related (59%) with more than half (n = 7/13 [53.8%] rhythm‐related including one ventricular and six atrial events. The low‐volume indications of deep vein thrombosis/pulmonary embolism and stroke/ transient ischemic attack evaluation also demonstrated comparatively high rhythm‐related events at 26.8% and 34.9% respectively. When the “Other” category (n = 9799 [13.6%]) was analyzed, the most common grouping was hypotensive disease states (n = 607 [<1%]) including gastrointestinal bleeding, sepsis/bacteremia, and pancreatitis among others. Hypotensive disease state was identified in 40 ERT activations (6.6%). Of these, noncardiac events were identified in 30 (75%).

**Figure 1:**
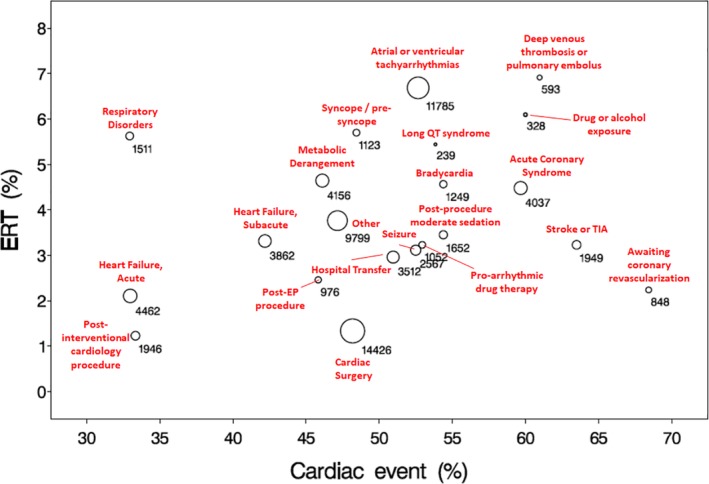
Bubble plot depicting volume for annotated telemetry indications along with the overall emergency response team (ERT) event rate on the Y axis, and cardiac‐related ERT event rate on the X axis. The size of each annotated indication bubble is proportional to the order volume. The right upper quadrant identifies patients with high overall and cardiac‐related event rates (eg, deep venous thrombosis/pulmonary embolism [DVT/PE]). The left upper quadrant identifies patients with high noncardiac ERT event rates (eg, respiratory disorders). The right lower quadrant identifies patients with high cardiac‐specific event rates (eg, awaiting coronary revascularization). The left lower quadrant represents overall lower risk

The results of bubble plot analysis measuring order volume in addition to overall and cardiac‐specific ERT activation event rates are shown in Figure 1. In this graph, the percentage of ERT activations is displayed on the Y axis with cardiac‐specific events on the X axis, thereby creating visual representation of the indication‐specific risk profile. For example, patients awaiting coronary revascularization appear in the lower right quadrant of this graph due to a low overall ERT event rate, but high proportion of cardiac events. Patients with deep venous thrombosis or pulmonary embolus feature both a high percentage of overall ERT and cardiac‐related events, suggesting concern for both respiratory and cardiovascular events. Conversely, patients undergoing telemetry monitoring for respiratory disorders appear in the left upper quadrant as having a high overall event rate, but a low proportion of cardiac events. The lowest risk indications therefore cluster towards the lower left quadrant. The size of each bubble indication is proportional to the number of telemetry orders generated in that category.

## 4 DISCUSSION

The key finding of this analysis is that hospitalized patients at highest risk for clinical deterioration due to both cardiac and noncardiac causes, as measured by ERT activation, can be identified by telemetry monitoring indication, and thereby prioritized in monitored bed resource constrained circumstances. This includes hospitals with a fixed number of telemetry capable stepdown beds, or those otherwise limited by availability of telemetry equipment or monitoring personnel. These data respond to a call for clinical evidence embedded within the 2017 update to the American Heart Association guidelines, which have been historically derived largely by expert opinion.^6^

The present analysis has identified high‐risk patients with noncardiac indications for telemetry monitoring including those with metabolic derangements, stroke evaluation, deep vein thrombosis/pulmonary embolism, seizure monitoring, and drug exposures (ie, alcohol, amphetamines). When compared to the 2017 Update to Practice Standards, there is agreement on the appropriateness of monitoring for metabolic derangements, stroke evaluation, and drug exposures. However, the document gives no recommendation for DVT/PE due to lack of evidence, and does not recommend seizure monitoring on the basis of available evidence. In the present analysis, stroke evaluation, deep venous thrombosis/pulmonary embolism and drug exposures were among the top yielding indications for cardiac‐related events. Therefore, the authors suggest there is now sufficient evidence to upgrade stroke evaluation and metabolic derangements to class I recommendations, and to consider adding a recommendation regarding DVT/PE in the selected cases where inpatient hospitalization treatment is required. Further studies are recommended with regards to seizure monitoring, as well as further delineation of the event rates with specific recreational drug and alcohol exposures, which were combined at the level of order entry in the present analysis, and thus cannot be separated. The authors suggest the methodology applied in Figure 1 provides a useful construct for assessing categories of monitored patients according to risk for cardiac and noncardiac clinical deterioration. While the present analysis was conducted in a high‐volume, high‐acuity hospital and referral center, these methods could easily be replicated and applied to community hospitals and a different spectrum of clinical conditions including those specified requiring further study. For example, the indication for seizure monitoring in our center was largely confined to a dedicated epilepsy unit where patients are admitted for provocative testing and monitored for vagally mediated bradycardia with seizure, and/or pro‐arrhythmic effects related to the use of anti‐epileptic agents with sodium channel blocking properties.

Careful review of these data suggests that the association between initial telemetry indication and clinical deterioration are inherently limited by selection forces in assigning a diagnosis and choosing an indication category. As an example, the ERT and cardiac event rates observed among patients with acutely decompensated heart failure and postcardiac intervention procedures seems disproportionately low when considering medical acuity in relation to the other monitored patient categories. However, it is important to consider that such patients are often clustered into highly specialized cardiac stepdown units where the threshold to trigger ERT activation may be higher for expected problems (ie, angina in a patient following coronary intervention) that nursing staff and covering physicians are trained and equipped to routinely manage. A similar problem occurring in a nonspecialized nursing unit (ie, angina occurring on a general medical ward) may be more likely to trigger an ERT activation, as this exceeds routinely delivered care in such a unit. Thus, considering the association between the initial telemetry indication and clinical outcomes is useful, but insufficient for comprehensive risk stratification. Future work should build upon this principle to create clinical decision support systems incorporating indication‐specific risk along with other parameters changing in real‐time such as alarms and vitals trending data to better hone attention towards a deteriorating patient within a given unit. This clinical decision support system could be paired with a patient's electronic health record to create a population management tool. Directing ERT resources to the highest risk patients may improve response time and potentially prevent further decompensation of patient's condition in the non‐ICU environment.

There are a number of important limitations in the present data set. The first caveat is that data derived from a single center with high‐acuity patients cannot be generalized to dissimilar hospital environments and patient populations. This is readily apparent in the uncommonly high volume of patients monitored following cardiac surgery. However, this limitation is at least partially mitigated by an impressive volume of monitored patients for noncardiac indications, including those with metabolic derangements, drug exposures, DVT/PE, and for stroke evaluation. Furthermore, publishing and reported these data represent an important first step to facilitate future analysis of community hospitals and across a spectrum of clinical acuity. Second, the lack of demographic data for the monitored patients in this study prohibits a more enriched multivariable analysis according to age, sex, or disease‐specific parameters. While perhaps amplifying selection bias, it also underscores the importance of foundationally grounding the need for telemetry monitoring using bedside clinical assessment within the framework of standardized indications. Associated outcomes data are lacking in this field, and unfortunately seldom reported. Thirdly, several indications categorically combine patients of varying pathophysiology such as in the case of recreational drug and alcohol exposures. Regrettably, our data set does not allow the separation of these exposures as they were combined at the point of order entry. With that said, this indication does thematically capture the risk associated with substance abuse, as recreational drug exposures and alcohol intoxication often occur concomitantly. Yet, each drug exposure likely carries distinct risk and the authors recommend further evaluation in future studies. This is also likely true in the moderate sedation postprocedure and heart failure categories whereby the timing, total dosage and choice of drugs administered are likely contributory to specific event rates. Again, dedicated study of these populations utilizing enriched patient‐specific data is needed.

## 5 CONCLUSION

High‐risk hospitalized patients for subsequent ERT activations can be identified by telemetry monitoring indication, and thereby prioritized when monitored bed resources are constrained according to concerns for cardiac, arrhythmia‐specific, and noncardiac clinical deterioration.

## CONFLICT OF INTEREST

The authors declare no potential conflict of interests.
